# Diversity and Prevalence of Coral Diseases in the Nearshore Regions of the Northern South China Sea

**DOI:** 10.1002/ece3.72562

**Published:** 2025-12-11

**Authors:** Shaotong Tu, Lan Qiu, Jingjing Zhang, Yisi Hu, Wen Yu, Hao Luo, Shichao Wei, Zhiwei Zhang, Fuwen Wei, Wenliang Zhou

**Affiliations:** ^1^ Center for Evolution and Conservation Biology Southern Marine Science and Engineering Guangdong Laboratory (Guangzhou) Guangzhou China; ^2^ CAS Key Laboratory of Animal Ecology and Conservation Biology, Institute of Zoology Chinese Academy of Sciences Beijing China; ^3^ Jiangxi Key Laboratory of Conservation Biology, College of Forestry Jiangxi Agricultural University Nanchang Jiangxi China

**Keywords:** coral bleaching, coral diseases, coral reef degradation, coral reefs, South China Sea

## Abstract

Coral reef ecosystems have rapidly degraded under the combined pressures of climate change and human activities, with diseases further eroding ecosystem function and habitat resilience. Over the past few decades, different coral diseases have been prevalent in all coral reef regions of the world. However, there are few reports on the prevalence of coral diseases in the South China Sea (SCS). Therefore, this study conducted a systematic survey of coral disease diversity and prevalence in the nearshore regions of the northern SCS, covering seven survey sites along the coastlines of Guangdong Province and Hainan Island. Six common coral diseases were identified: Pink‐Line Syndrome, Trematodiasis Inflammation, White Syndrome, Skeletal Eroding Band, Ulcerative White Spot, and Growth Anomalies. The results revealed the prevalence and regional distribution patterns of coral diseases in the nearshore regions of the SCS and further assessed the susceptibility of coral species and host specificity of the common diseases. Additionally, environmental parameters and stress‐related phenomena affecting corals were recorded, and generalized linear mixed models (GLMMs) were applied to identify variables significantly correlated with coral disease prevalence. The findings further emphasize the critical role of regional environmental heterogeneity in shaping the prevalence patterns of coral diseases, providing important scientific insights to support coral disease management and coral reef ecosystem conservation in the SCS.

## Graphical abstract

This study presents the first systematic survey of coral disease diversity and prevalence in the nearshore regions of the northern South China Sea. Six common diseases and eight stress‐related phenomena were documented across seven sites, revealing distinct regional patterns and host‐specific susceptibilities. Our findings highlight the influence of environmental heterogeneity on disease dynamics and offer crucial insights for coral reef conservation in the region.

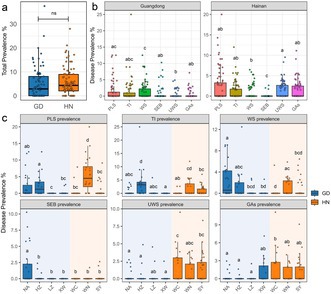

## 1 Introduction

Coral reef ecosystems, characterized by reef‐building corals as their foundational organisms, are unique ecological systems with exceptionally high biodiversity and play a critical role in marine ecosystems (Galand et al. 2023). These ecosystems not only provide diverse habitats and food resources for marine species but also offer valuable ecosystem services, as well as medicinal, esthetic, and cultural benefits to human society (Costanza et al. 2017). Since the Industrial Revolution, coral reefs have faced unprecedented mortality rates, further exacerbated by escalating human activities and global climate change (Morais et al. 2021). Specifically, the Caribbean and Indo‐Pacific regions, which are the primary zones of global coral reef distribution, have experienced declines in coral cover of 80% and 50%, respectively, over the past four decades (Morais et al. 2022). Coral diseases have become an increasingly critical global issue, with frequent outbreaks that further undermine the functional integrity and resilience of coral reef habitats (Vega Thurber et al. 2025). These outbreaks are often associated with significant coral mortality, driving key foundational species to critically low levels and hindering global restoration efforts (Randazzo‐Eisemann et al. 2022; Dehnert et al. 2024). However, our current understanding of coral diseases remains inadequate to overcome many of the challenges in coral reef conservation. The causes of most coral diseases, along with the mechanisms underlying key epidemiological aspects (e.g., pathology, transmission, dispersion, environmental drivers, modeling, and treatments), remain largely unknown (Vega Thurber et al. 2025).

In recent years, the global prevalence of coral diseases has increased substantially. As of 2022, more than 40 coral diseases had been documented worldwide, with 22 reported in the Atlantic and Caribbean, and 9 in the Indo‐Pacific, along with the combined West, South, and North Pacific regions, affecting over 200 species of reef‐building corals (Morais et al. 2022). Common coral diseases include Black Band Disease (BBD), White Band Disease (WBD), Yellow Band Disease (YBD), Brown Band Disease (BrB), Dark Spot Disease (DSD), Skeletal Eroding Band (SEB), Pink‐Line Syndrome (PLS), Growth Anomalies (GAs), Stony Coral Tissue Loss Disease (SCTLD), White Plague (WP), and White Syndrome (WS) (Morais et al. 2022; Vega Thurber et al. 2025; Kubomura et al. 2021). BBD, WBD, YBD, BrB, DSD, SEB, PLS and GAs are easily identifiable due to their distinctive lesions. In contrast, diseases such as SCTLD, WP, WS, and WBD are primarily characterized by tissue loss and exposure of the white coral skeleton; however, the specific pathogens or triggers responsible for these diseases remain unidentified. The absence of definitive diagnostic criteria complicates the diagnosis of these diseases.

The occurrence and transmission of coral diseases are widely recognized as the outcome of complex and dynamic interactions among the host, pathogens, and environmental parameters (Thrusfield 2015). Since the 1980s, although global efforts in the monitoring and assessment of coral diseases have continued to expand, progress in identifying causative agents, clarifying their associations with environmental drivers, and advancing other aspects of coral disease etiology remains limited (Bruckner 2015). This knowledge gap presents a significant challenge to the effective control and mitigation of coral diseases. Environmental stressors have been shown to significantly impair coral health, placing corals in a “suboptimal” or “sub‐healthy” physiological state that predisposes them to disease. Under such conditions, many microorganisms previously regarded as primary pathogens may instead act as opportunistic or non‐specific secondary colonizers that exploit weakened host defenses (Lesser et al. 2007). Evidence from long‐term field monitoring and both laboratory and in situ experiments has identified several environmental factors that contribute to disease onset or increase pathogen virulence, including elevated sea surface temperatures, increased solar irradiance, pollution, sedimentation, and algal overgrowth (Harvell 2002; Harvell et al. 2007).

The coral reefs of the South China Sea (SCS) are part of the Indo‐Pacific biogeographic province, located at the northern edge of the Coral Triangle, and represent an essential component of global coral reef systems (Allen 2007; Huang et al. 2008; Huang et al. 2025). Long‐term ecological monitoring has revealed that coral reefs in this region are undergoing rapid and extensive degradation, at a rate exceeding that of other major coral reef hotspots worldwide (Yu 2012). By 2015, live coral cover in the northern SCS (mainly Hainan Island, Weizhou Island, and Daya Bay) had already declined to approximately 10% (Qin et al. 2023; Li et al. 2024). Coral reef research in China began in the 1960s, primarily focusing on surveys of coral species and environmental characteristics. However, reports on coral diseases have remained scarce. Yang et al. (2014) documented an outbreak of Black Band Disease, dominated by *Cyanobacteria*, around Yongxing Island in the Xisha Archipelago, suggesting that the spread of this disease contributed to the rapid decline of coral reefs in the area. To date, no comprehensive study on coral disease outbreaks in the SCS has been conducted. The northern SCS is densely populated and subject to multiple anthropogenic pressures, including overfishing, coastal development, and watershed pollution. However, it remains unclear whether, and to what extent, these stressors influence the prevalence, types, and spatial distribution patterns of coral diseases. Therefore, this study aims to perform a comprehensive survey of coral diseases in the nearshore coral reef regions of the northern SCS, assessing their prevalence, identifying the main affected coral species, and exploring their spatial distribution patterns. Furthermore, it examines stress‐related phenomena linked to coral diseases and analyzes the primary drivers of disease outbreaks, thereby providing scientific evidence for coral disease management and the conservation of coral reef ecosystems in the SCS.

## 2 Methods

### 2.1 Survey Design

**FIGURE 1:**
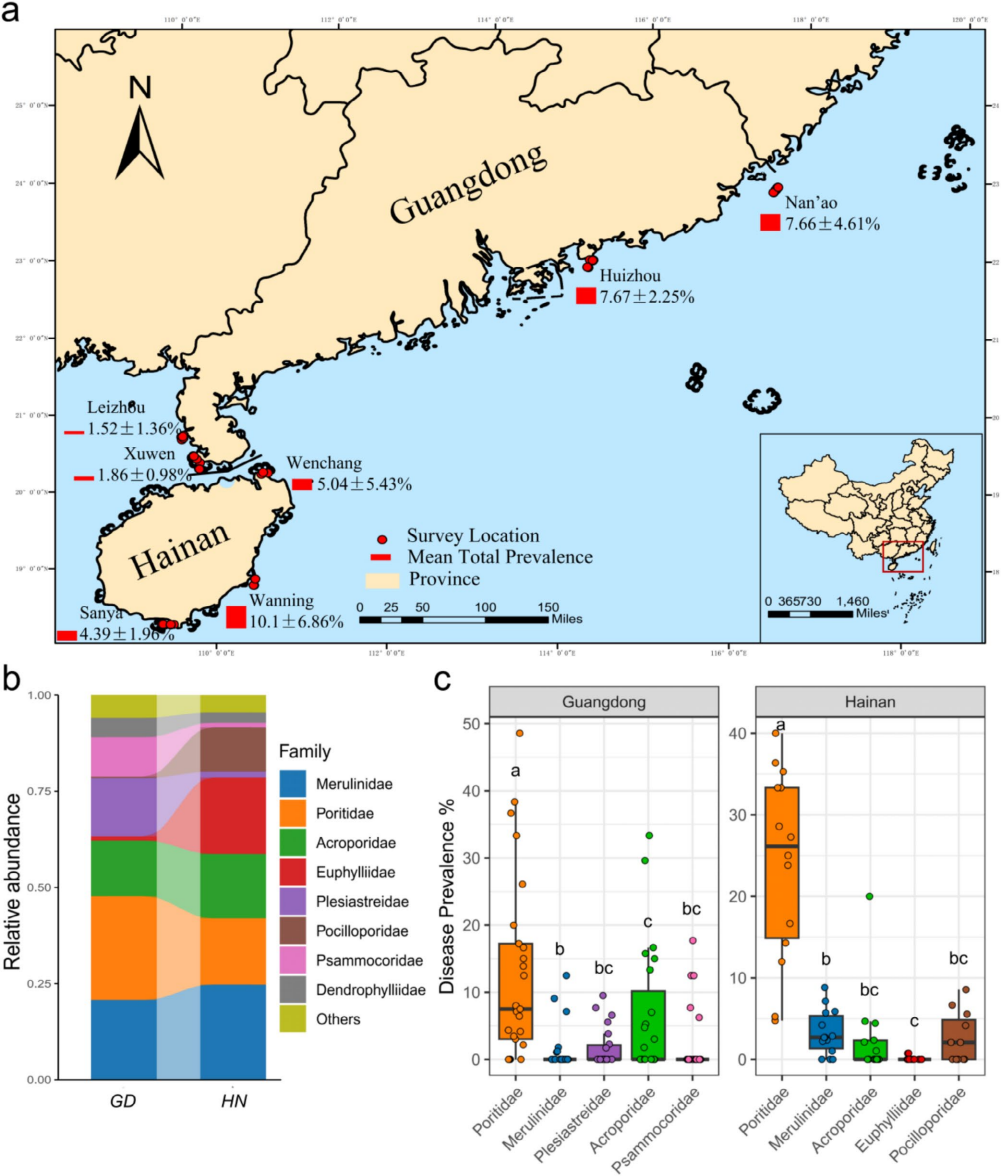
(a) Distribution of survey locations and total disease prevalence at each survey site along the coasts of Guangdong Province and Hainan Island; (b) Coral species composition and relative abundance at the family level; (c) Total disease prevalence of high‐abundance coral families along the coasts of Guangdong Province and Hainan Island. Lowercase letters indicate statistical differences among groups. Groups that share the same lowercase letter are not significantly different from each other, whereas groups with different letters differ significantly at *p* < 0.05.

This study aims to conduct a systematic investigation into the prevalence of coral diseases in the nearshore waters of the northern SCS. To achieve this, seven sea areas were selected along a latitudinal gradient spanning from Guangdong Province to Hainan Island. The survey sites included Nan'ao Nanpeng Archipelago (NA), Huizhou (HZ), Leizhou (LZ), and Xuwen (XW) in Guangdong Province, as well as Wenchang (WC), Wanning (WN), and Sanya (SY) along the Hainan coastline (Figure 1a). A total of 40 survey locations were surveyed between March and June 2024. During the survey, GPS coordinates were recorded for each site, and environmental parameters, including temperature (T), salinity (S), pH, turbidity (FNU), dissolved oxygen saturation (DO%) and concentration (DO, mg/L), were quantified using a YSI multi‐parameter instrument.

Following the standard protocols for coral disease surveys described by Raymundo et al. (2008), we conducted underwater belt‐transect surveys using SCUBA diving at depths of 3–10 m across fringing reefs, encompassing areas from the reef flat to the upper reef slope. The procedure was as follows: a transect line, 70–100 m long, was laid across the reef area, with 3–4 subplots established along the transect. Each subplot was 20 m long and 2 m wide (extending 1 m on either side of the transect line), covering a total area of 40 square meters per subplot. Subplots were delineated at 5 cm intervals along each transect (Figure S1). Within each subplot, photographs were taken to document all colonies of scleractinian corals with diameters greater than 5 cm. Colonies were included in the records only if more than 50% of their volume fell within the subplot. For densely growing colonies of the same species, their bases were closely examined to determine whether they were connected or fused. If the distance between colonies exceeded 5 cm and no physical fusion was observed, they were recorded as distinct colonies. The images collected during the surveys were carefully analyzed in the laboratory through manual classification to the genus level. This study employed the systematic taxonomic framework established by the World Register of Marine Species (WoRMS) Editorial Board, further refined by incorporating the contributions of Dai (2009) and Huang, Jiang, and Yuan (2021) to enhance taxonomic accuracy.

### 2.2 Coral Disease and Stress‐Related Phenomena Identification

**FIGURE 2:**
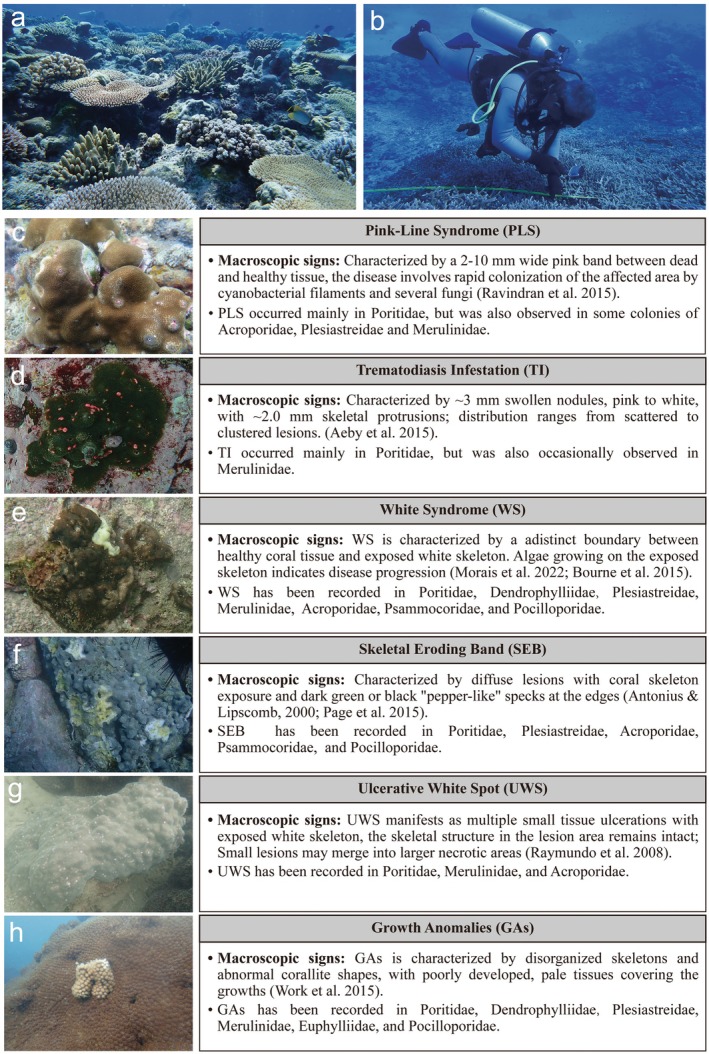
(a, b) Underwater photo of coral disease survey work; (c–h) Common coral diseases in the nearshore areas of the northern SCS, (c) Pink‐Line Syndrome (PLS), (d) Trematodiasis Inflammation (TI), (e) White Syndrome (WS), (f) Skeletal Eroding Band (SEB), (g) Ulcerative White Spot (UWS), (h) Growth Anomalies (GAs).

The diagnostic criteria for coral diseases based on macroscopic signs are shown in Figure 2c–h. In this study, pigment response was distinguished from Pink‐Line Syndrome (PLS) and Trematodiasis Inflammation (TI), and classified as a stress‐related phenomenon. Pigment response primarily referred to localized inflammatory reactions in coral tissues resulting from mechanical stresses induced by predation, aggressive overgrowth, boring organisms (e.g., *Ceraesignum maximum*, *Spirobranchus giganteus* ), Lithoglyptidae, and other unidentified factors (Figure S3a,b,d,h). Bleaching was defined as the partial or complete loss of symbiotic zooxanthellae in corals, which was carefully distinguished from the natural absence of zooxanthellae at growth tips or edges in newly formed coral tissue (Figure S3c). Sedimentation referred to the accumulation of sediments on coral surfaces that formed a visible layer (Figure S3d). Aggressive overgrowth described the encroachment of coral surfaces by turf algae, macroalgae, sponges, or other competing organisms (Figure S3e). Excess mucus was recorded when more than 20% of the coral surface was covered by mucus, which often appeared as a white film that trapped sediments or debris from the surrounding water (Figure S3d). Mechanical injury was characterized by fractures or injuries caused by external forces, carefully differentiated from predation‐related damage (Figure S3f). Predation was noted when corals exhibited feeding marks from fish, crown‐of‐thorns starfish, or gastropods (Figure S3g). Parasitism was documented when corals were infested by trematodes or boring organisms (Figure S3h). These coral stress‐related phenomena may occur simultaneously (Figure S3d,h).

### 2.3 Data Statistics and Analysis

Based on taxonomic identification and coral counts, the relative abundances of coral genera and families within each subplot and survey site were calculated, followed by alpha and beta diversity analyses (Spellerberg and Fedor 2003). Subsequently, the prevalence of coral diseases and associated stress‐related phenomena was quantified based on disease diagnoses and recorded stress‐related phenomena. The following statistical analysis procedure was applied to assess differences among survey sites across all datasets. Normality of the data was assessed using the Shapiro–Wilk test. Homogeneity of variances was evaluated with Levene's test for parametric data and the Fligner‐Killeen test for non‐parametric data. Parametric data satisfying the assumptions of normality and variance homogeneity were analyzed using one‐way ANOVA followed by Tukey–Kramer post hoc tests. When variances were unequal, Welch's ANOVA followed by Games‐Howell tests was applied. Non‐parametric data were analyzed by the Kruskal‐Wallis test with Dunn's post hoc comparisons.

Generalized linear mixed model (GLMM) was utilized to investigate the determinants of coral disease prevalence. The dependent variable was the disease status of corals (0 = healthy, 1 = diseased), with fixed effects comprising stress‐related phenomena and environmental parameters. To address multicollinearity, temperature (T) was excluded due to its strong correlation with salinity (S) and pH. The model also incorporated two random effects (Genus) and survey location (Sites) to account for hierarchical dependencies within the data. This comprehensive approach aimed to assess the impact of stress‐related phenomena and environmental parameters on coral disease prevalence and their explanatory power.

All data analyses in this study were conducted using R version 4.3.1. The R packages stats, FSA, car, and agricolae were employed to perform the statistical analyses outlined above. Alpha and Beta diversity analyses were carried out using the vegan package, while data visualization was performed using the ggplot2 package. Variable correlation analysis and visualization were conducted using the Hmisc and corrplot packages. Generalized linear mixed‐effects models were fitted using the R package lme4 to investigate the effects of fixed factors (e.g., stressors and environmental parameters) and random factors (e.g., survey locations or coral species) on coral disease prevalence. Model selection was subsequently conducted using the R package MuMIn, and AICc was applied to identify the optimal model set. Model averaging was then performed to evaluate the relative importance of the variables.

## 3 Results

### 3.1 The Diversity of Coral Species and Diseases in the Nearshore Regions of the Northern SCS

This study conducted surveys across seven survey sites along Guangdong Province and Hainan Island, establishing 40 survey locations and recording 152 subplots. A total of 5988 individual coral colonies were systematically photographed and cataloged (Table S1). Six common coral diseases were identified: Pink‐Line Syndrome (PLS), Trematodiasis Inflammation (TI), White Syndrome (WS), Skeletal Eroding Band (SEB), Ulcerative White Spot (UWS), Growth Anomalies (GAs) (Figure 2d–g). Among the seven survey sites, WN exhibited the highest overall prevalence of coral diseases, reaching 10.1% ± 6.68%, while LZ recorded the lowest prevalence, at just 1.52% ± 1.36% (Figure 1a). Family‐level taxonomic identification revealed that Merulinidae, Poritidae, and Acroporidae were the top three coral families in terms of relative abundance in the nearshore regions of the northern SCS. In the coastal waters of Guangdong Province, the other two most abundant families were Plesiastreidae and Psammocoridae, whereas in the coastal waters of Hainan Island, Euphylliidae and Pocilloporidae predominated (Figure 1b; Table S2). Additionally, significant differences in disease prevalence were observed among different coral families, with Poritidae exhibiting the highest prevalence in both Guangdong Province and Hainan Island (Figure 1c). Coral assemblages in Hainan exhibited greater species richness than those in Guangdong, and PCoA analysis revealed distinct community compositions between the two regions (Figure S2b,c; Table S3).

### 3.2 The Distribution Characteristics of Coral Diseases in the Nearshore Regions of the Northern SCS

**FIGURE 3:**
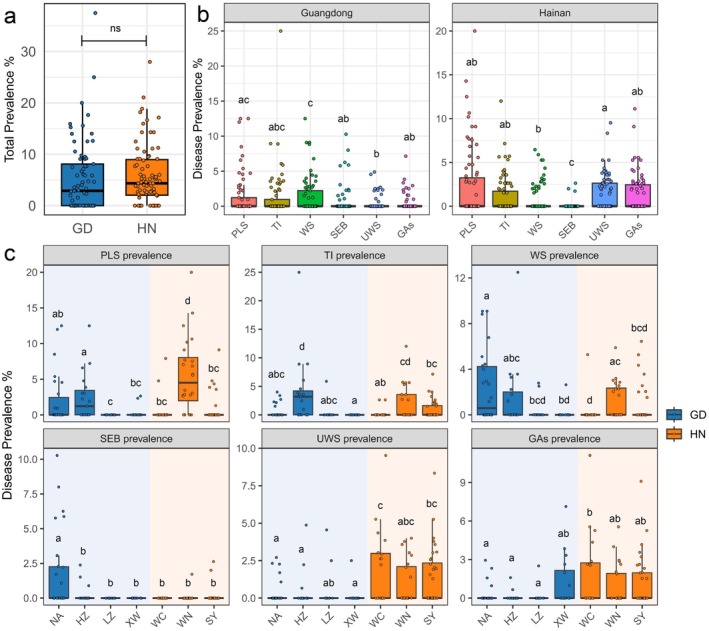
(a) Overall prevalence of coral diseases in Guangdong Province and Hainan Island; (b) Prevalence of six common coral diseases along the coasts of Guangdong Province and Hainan Island; (c) Prevalence of five common coral diseases at the seven survey sites. Lowercase letters indicate statistical differences among groups. Groups that share the same lowercase letter are not significantly different from each other, whereas groups with different letters differ significantly at *p* < 0.05.

**FIGURE 4:**
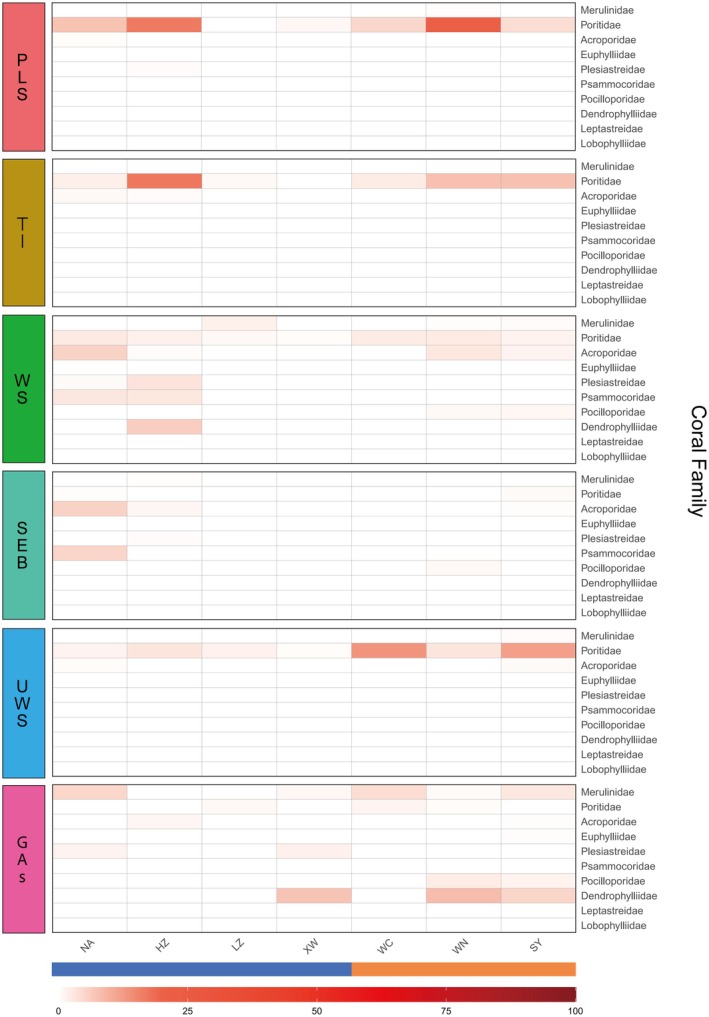
Heatmap of the specific prevalence of six common diseases in different coral families at each survey site.

Analysis of coral disease data revealed that, although the overall prevalence of coral diseases along the coasts of Guangdong Province (5.27% ± 6.69%) and Hainan Island (5.91% ± 5.79%) does not differ significantly (*p* = 1.30E‐01), marked differences were observed in disease composition between the two regions (Figure 3 and S5b; Table S4). Specifically, with the exception of PLS and TI, which were commonly detected along both coastlines, WS was predominantly found along the Guangdong coast, while UWS and GAs were more frequently recorded along the coast of Hainan Island (Figure 3b). Each coral disease exhibited distinct regional distributions across the survey sites (Figure S4). PLS and WS were primarily observed at the NA, HZ, and WN sites, with PLS reaching its highest prevalence in WN (5.61% ± 5.25%) and WS peaking in NA (2.41% ± 3.13%). TI was mainly concentrated at the HZ, WN, and SY sites. SEB was predominantly found at the NA site (1.79% ± 2.95%), the northernmost site surveyed, with minimal or no occurrence elsewhere. In contrast, UWS and GAs were mainly distributed at low‐latitude sites (Figures 3 and 4).

### 3.3 Host Preference of Coral Diseases in the Nearshore Regions of the Northern SCS

To further explore whether common coral diseases in the nearshore regions of the northern SCS exhibit host specificity, this study analyzed the specific prevalence of six common diseases in different coral families at each survey site. The results revealed that Poritidae was the most susceptible coral family to disease in the northern SCS, with all six common diseases recorded on this host. Among them, PLS (8.92% ± 17.61%), TI (6.19% ± 13.91%), and UWS (6.80% ± 15.02%) were primarily observed in Poritidae and exhibited relatively high prevalence. In contrast, these three diseases were only sporadically detected in other families such as Acroporidae, Merulinidae, and Plesiastreidae. WS had a broad host range, affecting multiple coral families, including Poritidae (1.88% ± 6.28%), Acroporidae (2.21% ± 7.75%), Plesiastreidae (0.77% ± 3.20%), and Psammocoridae (1.05% ± 3.99%). SEB was predominantly observed in Acroporidae (1.19% ± 4.98%) and Psammocoridae (0.62% ± 2.74%) at the NA site. GAs also affected multiple coral families in the northern SCS, with relatively high prevalence observed in Dendrophylliidae (5.22% ± 19.94%), Merulinidae (2.15% ± 7.06%), and Pocilloporidae (1.64% ± 6.01%) (Figures 4 and S5a; Table S6).

### 3.4 The Correlation Between Common Coral Diseases, Stress‐Related Phenomena, and Environmental Parameters

This study identified eight common stress‐related phenomena affecting corals in nearshore reef regions of the northern SCS, including pigment response, bleaching, sedimentation, aggressive overgrowth, excess mucus, mechanical injury, predation, and parasitism (Figure S3a–h). Data analysis of coral stress‐related phenomena in the nearshore regions of the northern SCS revealed that sedimentation, aggressive overgrowth, excess mucus and parasitism were significantly more prevalent along the coast of Guangdong Province compared to Hainan Island. No significant differences were observed between the two regions for the remaining three stress‐related phenomena (Figures S6 and S7; Table S5).

In the total prevalence model, the fixed‐effects analysis revealed that pigment response, bleaching, aggressive overgrowth, predation, parasitism, and salinity were significantly positively associated with coral disease prevalence, while stress‐related phenomena also showed significant associations with the prevalence of individual disease types (Table 1). Notably, WS exhibited significant positive correlations with the largest number of stress‐related phenomena, followed by PLS and TI, suggesting that complex pathogenic and transmission mechanisms may underlie the prevalence of these diseases. In addition, environmental parameters were also closely correlated with coral disease prevalence. For instance, salinity showed a significant positive correlation with disease prevalence in both SEB and UWS, whereas pH and FNU displayed opposite correlation patterns in WS, SEB, and UWS. These findings suggest that alterations in environmental parameters may influence coral disease dynamics through various pathways, depending on the ecological conditions (Table 1).

**TABLE 1:** Results of the generalized linear mixed model (GLMM) analysis on the key driving factors of coral diseases.

		Estimate	Std. error	*z* Value	Pr(> |*z*|)	Sig.
Total prevalence	(Intercept)	−4.75153	0.36317	−13.083	< 2e−16	***
	Pigment response	5.04576	0.42194	11.958	< 2e−16	***
	Bleaching	1.14250	0.29585	3.862	0.000113	***
	Aggressive overgrowth	0.79246	0.15103	5.247	1.54e−07	***
	Predation	1.05167	0.21156	4.971	6.66e−07	***
	Parasitism	0.70449	0.14492	4.861	1.17e−06	***
	S	0.25467	0.10176	2.503	0.012326	*
	FUN	−0.37541	0.14480	−2.593	0.009525	**
PLS	(Intercept)	−12.9422	2.6780	−4.833	1.35e−06	***
	Pigment response	0.4863	0.2400	2.026	0.04274	*
	Aggressive overgrowth	1.7688	0.3093	5.718	1.08e−08	***
	Parasitism	1.0189	0.2797	3.643	0.00027	***
TI	(Intercept)	−10.335598	2.250897	−4.592	4.4e−06	***
	Sedimentation	0.641924	0.297749	2.156	0.03109	*
	Predation	0.752331	0.333847	2.254	0.02423	*
	Parasitism	0.968195	0.316874	3.055	0.00225	**
WS	(Intercept)	−5.28460	0.44663	−11.832	< 2e−16	***
	Pigment response	−2.38206	1.03905	−2.293	0.02188	*
	Bleaching	1.67072	0.42387	3.942	8.1e−05	***
	Predation	0.89942	0.37537	2.396	0.01657	*
	Parasitism	0.75902	0.30491	2.489	0.01280	*
	PH	0.41451	0.19448	2.131	0.03306	*
	FUN	−1.09873	0.41311	−2.660	0.00782	**
SEB	(Intercept)	−6.7019	0.7844	−8.543	< 2e−16	***
	Predation	1.3703	0.4078	3.360	0.000778	***
	S	1.2205	0.5783	2.111	0.034797	*
	PH	0.8230	0.3907	2.107	0.035138	*
	FNU	−3.9291	1.4657	−2.681	0.007347	**
UWS	(Intercept)	−10.7443	2.2066	−4.869	1.12e−06	***
	S	0.8040	0.3665	2.194	0.02824	*
	PH	−0.6328	0.2785	−2.272	0.02308	*
	FUN	0.9415	0.3179	2.962	0.00306	**
GAs	(Intercept)	−6.1496	0.6736	−9.129	< 2e−16	***
	Bleaching	2.4408	0.5710	4.275	1.91e−05	***
	Mechanical injury	2.3477	0.8101	2.898	0.00375	**

*Note:* Asterisks indicate statistical significance: * *p* < 0.05, ** *p* < 0.01, and *** *p* < 0.001.

## 4 Discussion

Coral disease research worldwide has primarily focused on the Caribbean (34%) and the Indo‐Pacific (28.7%), which are the two main hotspots for coral diseases (Morais et al. 2022; RuizMoreno et al. 2012). This study represents the first systematic and quantitative assessment of coral disease diversity and prevalence in the nearshore regions of the northern SCS. The survey results indicate that the prevalence of coral diseases in the nearshore regions of northern SCS ranges from 1.52% to 10.1%, aligning with data from surrounding areas of the SCS and the broader Indo‐Pacific coral reef regions, including Taiwan (2.12%–8.58%), Okinawa (3.6%–9.7%), the Philippines (5.09%–11.6%), the Australian Solitary Islands (6.21%–13.58%), and Heron Island (1.9%–9.0%) (Willis et al. 2004; Raymundo et al. 2005; Haapkylä et al. 2010; Weil et al. 2012; Huang, Hwang, et al. 2021). The six common coral diseases identified in this survey have been previously reported in the surrounding waters of the SCS (Kubomura et al. 2021; Huang, Hwang, et al. 2021). Further analysis highlighted pronounced spatial variation in both the prevalence and types of common coral diseases among survey sites in the northern SCS (Figure 3b,c). This observation aligns with previously documented spatial patterns in coral disease distribution across the Indo‐Pacific region (Myers and Raymundo 2009; Haapkylä et al. 2010; Couch et al. 2014; Huang, Hwang, et al. 2021), underscoring the significant spatial heterogeneity of coral disease dynamics within this region.

To enhance the accuracy and scientific rigor of the survey results, this study provided a detailed distinction among PLS, TI, and pigment response, which are commonly observed in Poritidae and share similar macroscopic features, such as pink or bluish‐purple linear, punctate, or blotchy abnormal tissues. PLS and TI are classified as infectious coral diseases caused by pathogenic microorganisms or parasites. Among them, PLS exhibited the highest prevalence in the northern SCS, consistent with observations from nearby Okinawajima Island, Japan (Kubomura et al. 2021). Previous studies have implicated *Phormidium valderianum* as the causative pathogen of PLS in *Porites lutea* from the National Facility for Marine Cyanobacteria, Trichy, India (Anagnostidis and Komárek 1988). However, pathogens responsible for the same coral disease may vary significantly across geographic regions and host species, highlighting that diseases showing similar gross signs may have distinct etiologies and emphasizing the need for region‐ and host‐specific pathogen identification to improve diagnostic accuracy (Morais et al. 2022). The pathogen of TI has been implicated as *Podocotyloides stenometra* Prichard, primarily affecting *Porites* spp. (Aeby 2015). Our results demonstrate that sedimentation and predation are significantly positively correlated with the prevalence of TI. This finding indicates that sediment accumulation and predatory activity may create favorable conditions for the survival and transmission of the pathogen. Previous studies have also reported that coral predators (i.e., corallivores), such as butterflyfish, may selectively feed on TI lesions and act as intermediate hosts facilitating pathogen transmission, a behavior that further reinforces the positive association between predation and the prevalence of TI (Martin et al. 2018). Pigment response, in particular, has been identified as an inflammatory response in corals, acting as a defense mechanism against the aggressive overgrowth of algae, neighboring corals, and other organisms. It is also believed to be closely associated with stress induced by mechanical damage, chemical interference, and various environmental stressors (Palmer et al. 2009; Benzoni et al. 2011; D'Angelo et al. 2012; Ramesh et al. 2019; Ramesh et al. 2023). Accordingly, in this study, pigment response was classified as a stress‐related phenomenon rather than a coral disease. Notably, BDD, one of the most common and highly transmissible coral diseases in the Indo‐Pacific region, predominantly infects *Acropora* spp., *Montipora* spp., and *Leptoseris* spp., but has not been recorded in the nearshore regions of northern SCS (Johan et al. 2016; Morais et al. 2022; Pribawastuti et al. 2024). This phenomenon may be related to the relatively low abundance of these susceptible hosts in the region (Figure S2). Additionally, studies from the Great Barrier Reef suggest that the prevalence of BDD is higher in offshore areas with reduced human interference, a pattern also observed in Taiwan and Japan (Page and Willis 2006; Weil et al. 2012; Huang, Hwang, et al. 2021), which further explains the absence of BDD in the nearshore regions of northern SCS.

Among the six common coral diseases identified in the northern SCS, WS characterized by tissue loss, exhibits a broad host range, with host preferences in the northern SCS resembling those in nearby regions like Taiwan (Huang, Hwang, et al. 2021). In recent years, WS has also been documented in several coral reef areas of the Indo‐Pacific region (Bourne et al. 2016). However, the pathogenic agent behind WS remains unidentified, with bacteria, eukaryotes, and viruses potentially causing different forms of tissue loss in various coral species (Pollock et al. 2016; Morais et al. 2022). SEB is one of the most prevalent and host‐general diseases in the Indo‐Pacific region (Page and Willis 2007; Willis et al. 2004; Bises et al. 2024), with a significantly higher prevalence at the NA site compared to other sites, closely associated with predation (Figure 3c; Table 1). Corallivores at the NA site may act as vectors for the SEB pathogen *Halofolliculina corallasia*, facilitating its transmission (Montano et al. 2020). UWS and GAs were primarily observed at low‐latitude survey sites (Figure 3c). Although the pathogenic factors behind UWS remain unclear, its aggregated prevalence and experimental evidence showing that healthy corals are infected by UWS‐infected corals directly support the conclusion that UWS is a contagious disease (Raymundo et al. 2003; Bourne et al. 2016). The prevalence of UWS is associated with several environmental parameters (S, pH and FNU), suggesting that the physicochemical properties of seawater along the coast of Hainan Island may provide favorable conditions for the spread of this disease. The prevalence of GAs was significantly associated with mechanical damage. Previous studies have suggested that the progression of GAs involves host genes associated with tumor formation (Ricci et al. 2022). We hypothesize that corals may activate repair mechanisms following mechanical injury to restore damaged skeletal structures and tissues, and that dysregulation of these processes could subsequently disrupt normal skeletal growth. Additionally, abnormal coral skeletons may exhibit lower structural integrity, potentially increasing their susceptibility to further mechanical injury, thus reinforcing the association between GAs and mechanical damage (Domart‐Coulon et al. 2006).

Coral stress‐related phenomena not only reflect corals' responses to environmental pressures but also reveal the characteristics of regional environmental stressors. Generalized Linear Mixed Model (GLMM) analysis indicates that five stress‐related phenomena (pigment response, bleaching, aggressive overgrowth, predation, and parasitism) significantly increase the prevalence of coral diseases in the nearshore regions of the northern SCS (Table 1). Although the current evidence is insufficient to establish a causal relationship between stress‐related phenomena and coral disease occurrence, the results, when interpreted in the context of previous studies, may suggest several plausible ecological mechanisms. Firstly, during coral bleaching, the symbiotic relationship between corals and zooxanthellae is disrupted (Muscatine and Porter 1977; Hu et al. 2024), which not only disrupts their energy flow and material cycling, but also reduces their immune function and increases their disease susceptibility to pathogens (Hu et al. 2021). Secondly, the development of coastal areas has led to large amounts of sediment, suspended particulate matter and pollutants being transported to the marine environment through rivers and runoff (Fabricius 2005), which has promoted the eutrophication of seawater and provided a rich nutritional basis for the growth of algae (Lü et al. 2016). It may also create favorable conditions for the outbreak of coral reef pests, such as corallivores coral parasites (Bruno et al. 2003; Vega Thurber et al. 2014). The aggressive overgrowth of algae and sponges not only competes with corals for limited survival resources, but also weakens the immune function of corals by compromising the photosynthesis of coral symbiotic algae (Marubini and Davies 1996). The Guangdong coast is closer to the continental shelf and is more affected by landborne inputs, so it is not difficult to understand that it has a higher rate of aggressive overgrowth and parasitism than Hainan Island (Figure S6). Lastly, coral predators and parasitic organisms inflict physical damage on corals by consuming their mucus, external soft tissues, or skeletons, thereby creating pathways for pathogenic microorganisms to invade and increasing coral susceptibility to pathogens (Cole et al. 2008; Rotjan and Lewis 2008; Nicolet et al. 2013; Hoeksema et al. 2019). Furthermore, corallivores may also act as vectors for pathogens, facilitating disease transmission (Sussman et al. 2003; Martin et al. 2018).

Along with stress‐related phenomena, environmental parameters such as salinity, pH, and turbidity (FNU) were also significantly correlated with coral disease prevalence, indicating that fluctuations in seawater physicochemical conditions likewise play an important role in the occurrence and transmission of coral diseases. Specifically, elevated salinity can induce osmotic stress and disrupt the microbial community structure of the coral holobiont, thereby creating favorable conditions for opportunistic pathogenic infections (Röthig et al. 2016; Randle et al. 2020). Interestingly, the relationship between pH and coral disease prevalence varied among models, implying that the effects of pH on disease dynamics may be bidirectional and context‐dependent, shaped by both environmental background and pathogen sensitivity. A decline in pH can impair coral calcification and immune defenses and further destabilize the host–symbiont microbial equilibrium, thus facilitating pathogen invasion (Meron et al. 2010; Crook et al. 2013). Conversely, the growth and virulence of certain pathogens decrease under acidified conditions, suggesting that lower pH may, in some cases, suppress disease development (Muller et al. 2017). Recent evidence also emphasizes that pH variability, rather than its mean value, may represent a critical factor influencing the stability of coral holobionts and their susceptibility to disease (Tanvet et al. 2023). In this study, turbidity (FNU) exhibited a significant negative correlation with the prevalence of several coral diseases. Multiple lines of evidence indicate that moderate turbidity can attenuate incident light and buffer light–thermal synergistic stress during periods of elevated temperature, thereby reducing coral bleaching severity and subsequent disease susceptibility (Sully and Woesik 2020; Carlson et al. 2022). However, it is important to note that anthropogenically induced high suspended sediments and dredging activities markedly increase disease risk and inhibit coral reproduction and larval settlement, indicating that the effects of turbidity are threshold‐dependent and highly context‐specific (Pollock et al. 2014).

Overall, this study revealed pronounced regional differences in coral disease types and distribution patterns between the coasts of Guangdong and Hainan, which are likely linked to spatial variations in environmental conditions, oceanographic processes, and anthropogenic influences. Therefore, future research and monitoring programs should place greater emphasis on documenting changes in environmental parameters and the occurrence and progression of stress‐related phenomena. When early signs of these stressors intensify, targeted mitigation and management actions should be implemented to facilitate early warning and prevention. It should be acknowledged that the present study was based on a single field survey, and the data represent coral disease prevalence at one temporal snapshot. Given that coral disease prevalence can vary considerably over short time scales, future investigations should incorporate long‐term, time‐series observations to verify and expand upon the current findings. In view of these limitations, we advocate for the establishment of a long‐term, systematic coral disease monitoring framework in the northern SCS. Such sustained monitoring would generate temporally resolved datasets to improve understanding of the seasonal and interannual variability of coral diseases, elucidate their potential environmental drivers, and provide critical baseline information to support future reef management and conservation strategies.

## 5 Conclusion

This study presents the first systematic survey of coral disease prevalence in the nearshore regions of the northern SCS, identifying six common coral diseases. Our results indicate that coral disease prevalence is comparable between the coasts of Guangdong Province and Hainan Island. However, individual diseases exhibit distinct regional distribution patterns. Poritidae was the most susceptible coral family in the surveyed region, showing high prevalence of PLS, TI, WS, and UWS. Among these, PLS was most prevalent, highlighting the need for targeted research and continued monitoring. Five stress‐related phenomena (pigment response, bleaching, aggressive overgrowth, predation, and parasitism) were significantly positively associated with coral disease prevalence, underscoring the critical role of regional environmental heterogeneity in shaping the spatial patterns of coral diseases. Although the etiological agents and primary epidemiological mechanisms of most coral diseases remain unclear, the present findings provide new insights for future investigations. We plan to conduct long‐term monitoring of coral diseases, environmental parameters, and stress‐related phenomena in the future, with the aim of achieving early warning and mitigation of coral disease outbreaks and transmission in the nearshore regions of the northern SCS, thereby contributing to efforts to alleviate the ongoing degradation of coral reef ecosystems in this region.

## Author Contributions

**Shaotong Tu:** data curation (lead), formal analysis (lead), investigation (equal), visualization (lead), writing – original draft (lead). **Lan Qiu:** formal analysis (supporting), investigation (equal). **Jingjing Zhang:** data curation (equal), visualization (supporting). **Yisi Hu:** data curation (equal). **Wen Yu:** investigation (equal). **Hao Luo:** investigation (equal). **Shichao Wei:** investigation (equal). **Zhiwei Zhang:** investigation (equal). **Fuwen Wei:** conceptualization (equal), funding acquisition (equal), project administration (equal), writing – review and editing (equal). **Wenliang Zhou:** conceptualization (equal), funding acquisition (equal), investigation (equal), project administration (equal), writing – review and editing (equal).

## Funding

This work was supported by the Ministry of Science and Technology of China (2021YFF0502800), the Guangdong Science and Technology Department (2021QN02H103, 2023A1111110001, 2025B1212050002), the Forestry Administration of Guangdong Province (SLYJ2023B4004), and the PI Project of Southern Marine Science and Engineering Guangdong Laboratory (Guangzhou) (GML2020GD0804, GML2022GD0804).

## Conflicts of Interest

The authors declare no conflicts of interest.

## Supporting information


**Data S1:** ece372562‐sup‐0001‐DataS1.pdf.

**Data S2:** ece372562‐sup‐0002‐DataS2.xlsx.

## Data Availability

All data supporting the findings of this study are included in the manuscript and its Supporting Information.
