# Supplementary material for: Diversity and Prevalence of Coral Diseases in the Nearshore Regions of the Northern South China Sea
Source: Ecol Evol. 2025 Dec 11;15(12):e72562. doi: 10.1002/ece3.72562 (PMC12696479; doi:10.1002/ece3.72562)
Supplement: Supplementary file 1 — Data S1: ece372562‐sup‐0001‐DataS1.pdf. [file ECE3-15-e72562-s002.pdf]

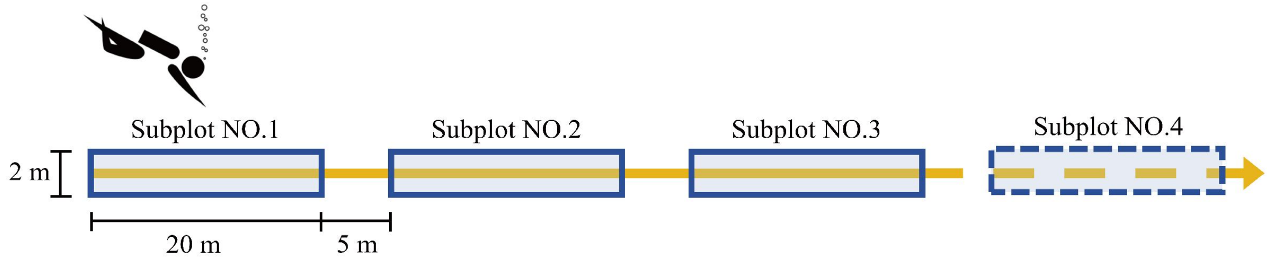

Fig. S1 Schematic of the coral disease belt-transect survey method.

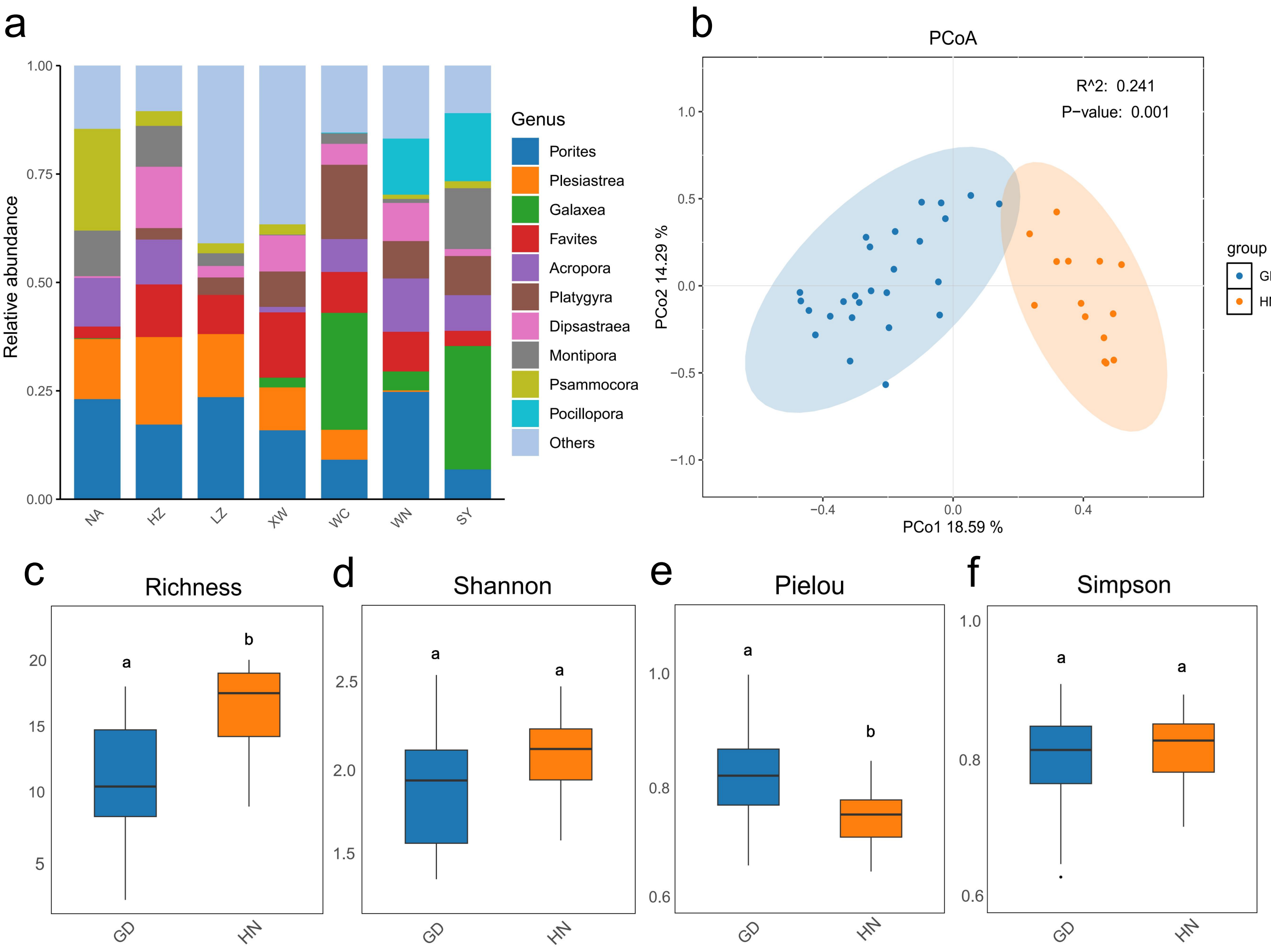

Fig. S2 a:Coral species composition and relative abundance at the genus level, **b**: Principal Coordinates Analysis (PCoA) of coral species composition along the coasts of Guangdong Province and Hainan Island; **c-f**: Alpha diversity analyses of coral species along the coasts of Guangdong Province and Hainan Island.

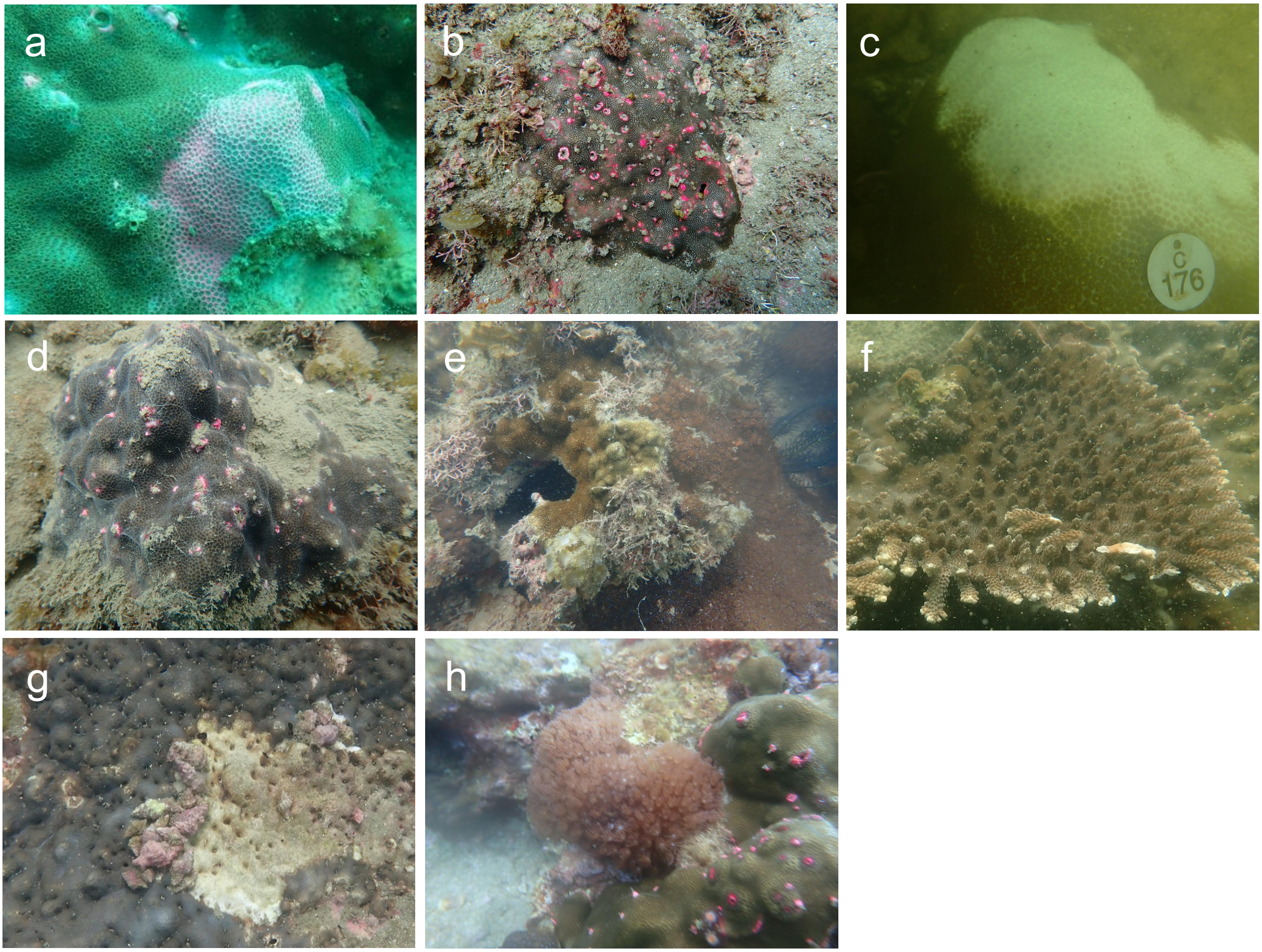

Fig. S3 Common coral stress-related phenomena, **a**: Pigment response (unknown factors), **b:** Pigment response caused by boring organisms, **c:** Bleaching, **d:** Sedimentation, pigment response and excess mucus, **e:** Algal aggressive overgrowth, **f:** Mechanical injury, **g:** Predation by gastropods, **h:** Parasitism and pigment response.

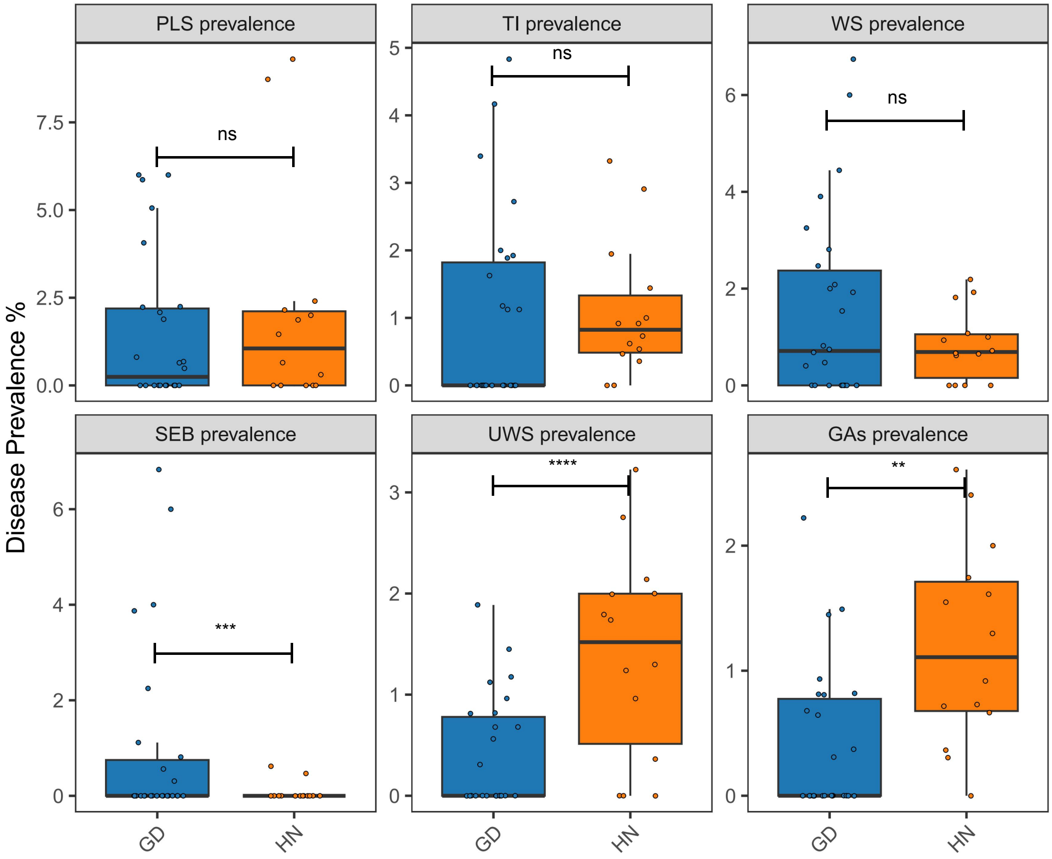

Fig. S4 Prevalence of six common coral diseases along the coasts of Guangdong Province and Hainan Island.

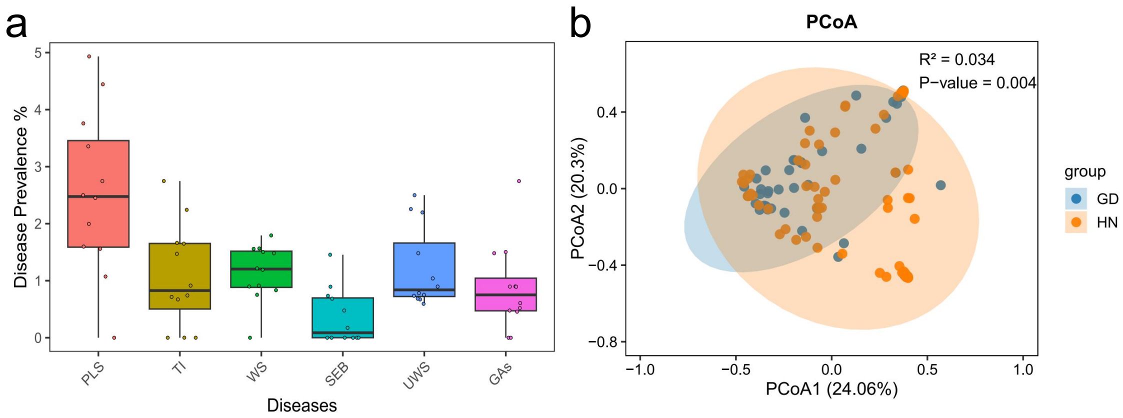

Fig. S5 a:Prevalence of six common coral diseases along the coasts of northen SCS; **b**: Principal Coordinates Analysis (PCoA) depicting variation in coral disease composition between Guangdong nd Hainan.

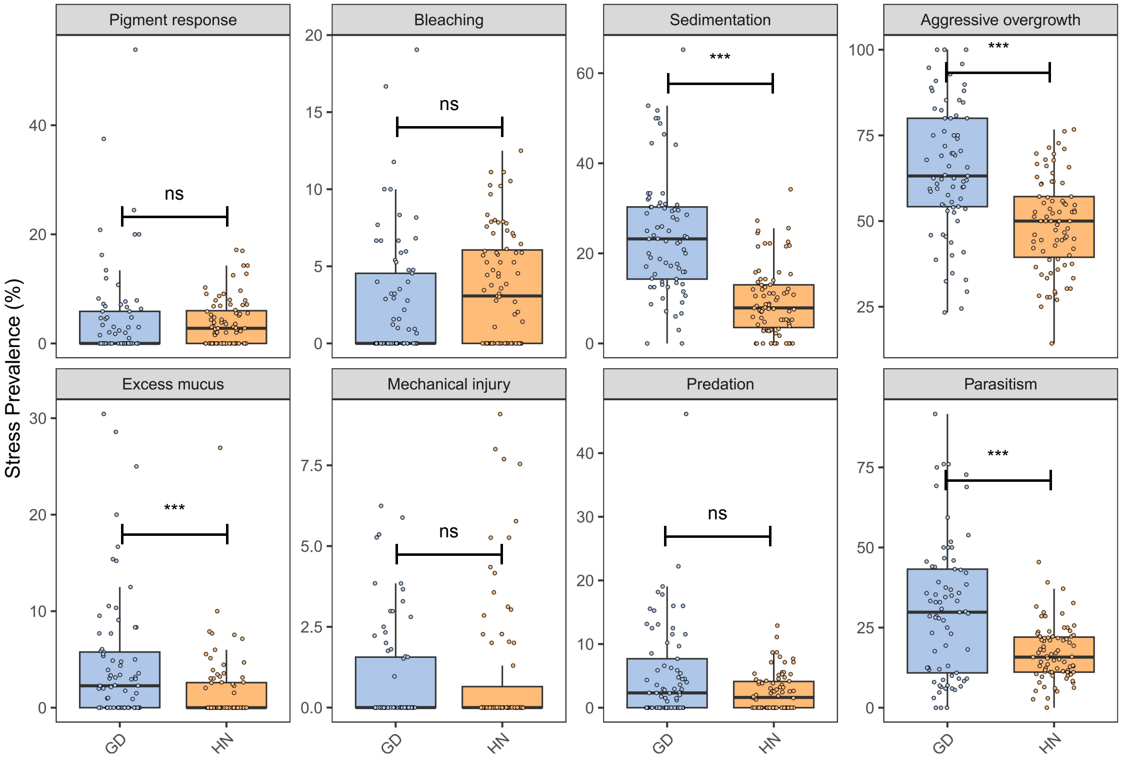

Fig. S6 Prevalence of eight common coral stress-related phenomena along the coasts of Guangdong Province and Hainan Island.

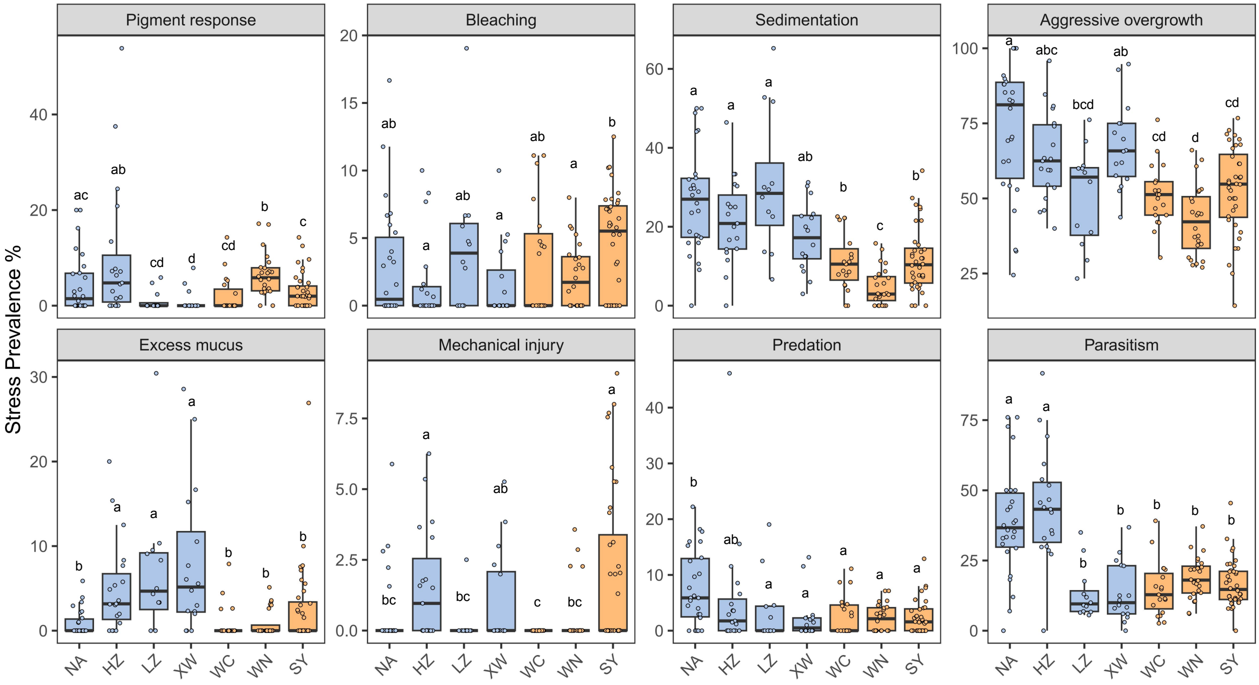

Fig. S7 Prevalence of eight common coral stress-related phenomena at seven survey sites.
